# Dietary insulin index, dietary insulin load and dietary patterns and the risk of metabolic syndrome in Hoveyzeh Cohort Study

**DOI:** 10.1038/s41598-024-52263-5

**Published:** 2024-01-23

**Authors:** Leila Elyasi, Fatemeh Borazjani, Kambiz Ahmadi Angali, Seyed Ahmad Hosseini, Nader Saki

**Affiliations:** 1 Nutrition and Metabolic Disease Research Center, Clinical Sciences Research Institute, Ahvaz Jundishapur University of Medical Sciences, Ahvaz, Iran; 2 Department of Biostatistics, School of Health Sciences, Ahvaz Jundishapur University of Medical Sciences, Ahvaz, Iran; 3 Department of Otolaryngology, Head and Neck Surgery, Hearing Research Center, Ahvaz Jundishapur University of Medical Sciences, Ahvaz, Iran; 4 Social Determinant of Health, Ahvaz Jundishapur University of Medical Sciences, Ahvaz, Iran; 5 Department of Nutrition, School of Allied Medical Sciences, Ahvaz Jundishapur University of Medical Sciences, Ahvaz, Iran

**Keywords:** Nutrition, Epidemiology

## Abstract

Postprandial insulin secretion has been associated with metabolic disorders such as hyperlipidemia and type 2 diabetes. Therefore, we aimed to explore the relationship between dietary insulin indices and dietary pattern with the risk of Metabolic Syndrome (MetS). The participants of the present cross-sectional study were included among the individuals who participated in the Hoveyzeh Cohort Study (HCS). A total of 3905 Iranian adults, aged 35–70 years, are included in the current analysis. The Food Frequency Questionnaire (FFQ) is used to calculate the dietary Insulin Index (DII), Insulin Load (DIL), and dietary pattern. Dietary pattern was derived using Reduced-Rank Regression (RRR) based on intake of protein (g/day), fiber (g/day), fat (g/day), magnesium (mg/day), and dietary insulin index were considered as response variables. The Generalized Linear Model was used to obtain the odds ratio (OR) and 95% confidence interval (CI) for MetS based on gender, while considering quartiles of DIL, DII scores, and dietary pattern, adjusted for potential confounders. The mean ± SD of age and BMI of the participants in the top quartile of DIL were 45.72 ± 8.05 years and 28.25 ± 5.02 kg/m^2^, respectively. The mean ± SD of DII was 40.53 ± 4.06 and the mean ± SD of DIL was 117,986.1 ± 30,714.06. A significant positive association was observed between DIL and MetS in women after adjusting for confounding factors (OR: 1.51; 95% CI 1.16; 1.96). No significant association was seen between DIL, DII, and MetS among men. A derived dietary pattern characterized by high intakes of fruits, sugar, sweet deserts, Whole Grains, and dairy was associated with an increased risk of MetS in adjusted model2 among women (OR: 1.41; 95% CI 1.13; 1.75) and men in the same model (OR: 2.09; 95% CI 1.35; 3.21).However, the final model was significant just for men (OR: 2.08; 95% CI 1.35; 3.21) and not for women (OR: 1.24; 95% CI 0.96; 1.60). Our findings showed that adherence to a diet with a high insulin load can increase the risk of MetS in women. In addition, a derived dietary pattern by RRR indicated that a diet rich in fruits, sugar, sweet deserts, whole Grains, and dairy is related to increased risk of MetS in both men and women.

## Introduction

Metabolic syndrome (Mets) is a complex cluster of interrelated factors that occur together, including abdominal obesity, dyslipidemia, increased blood pressure, and elevated blood glucose levels^1^. Despite the diagnostic criteria used, the prevalence of metabolic syndrome has been increasing in recent decades. The prevalence of metabolic syndrome and its components varies in different populations and ranges from 20 to 25% in the adult population^2,3^. A recent systematic review and meta-analysis of 472,401 Iranian people reported that 26% have MetS, which is more common in females 34% vs. males 22%^4^. As MetS is a multifactorial disorder, several factors are involved in the etiology of this disease such as socio–economic status, psychosocial stress, genetic factors, sedentary lifestyle, and dietary factors^5^. Better MetS prevention requires gender considerations due to sex differences influenced by aging and the environment^6^.

Meanwhile, the role of diet as a modifiable factor in the management of metabolic syndrome is important. Previous studies have often focused on the impact of single foods or dietary components on metabolic syndrome^7,8^. While, current investigations propose dietary patterns to assess the effect of diet on diseases due to the interactive and synergistic effects of dietary compounds on each other and diseases^9,10^. In the meta-analysis with the inclusion of forty observational studies, the “Healthy” pattern was associated with reduced risk of MetS in both genders and Eastern countries, particularly in Asia^11^.

Eating veggies, fruits, poultry, fish, and whole grains lowers Metabolic Syndrome risk. Eating red meat, animal fats, refined grains, eggs, and sweets increases risk^11,12^. The researchers investigated the effects of foods on postprandial insulin secretion to prevent and manage conditions such as hyperlipidemia and type 2 diabetes^13^. In this context, great attention has been given to carbohydrate as the major stimulus for insulin secretion due to its effect on blood glucose^14^. Despite this fact, postprandial insulin secretion is also affected by some other factors such as fructose, certain amino acids, fatty acids and gastrointestinal hormones^15,16^.

Insulin indices, such as the dietary insulin index (DII) and dietary insulin load (DIL), have been developed to measure insulin secretion in response to different foods, regardless of macronutrient composition^17^. The Dietary Insulin Index evaluates a food's insulin index value based on energy content and frequency of consumption. It's a more accurate indicator of insulin response than just looking at carbohydrate content^18^.

The association between insulin indices and some metabolic disorders including type 2 diabetes, low level of high-density lipoprotein (HDL) cholesterol and cardiovascular disease (CVD) has been previously reported^13,19,20^. Studies on the relationship between dietary insulin indices and MetS in Iran have produced conflicting results. According to their report, weight gain, regardless of DIL, heightened the chances of MetS in both genders. However, when weight remained stable, low DII and DIL had a more positive impact on women than on men. Higher DIL and DII were strongly related to an increased odds ratio of metabolic unhealthy obesity in Iranian adolescents. In women, high DIL and DII were significantly associated with increased odds of developing MetS. In men, moderate DIL was associated with increased odds of MetS. While, a study reported that participants in the highest quartile of DII and DIL had insignificantly lower odds of MetS. Another study found that hs-CRP mediates the association between DIL and MetS. Additionally, DII has a weak association with the risk of MetS^21–25^.

Recent research evidence suggests an inverse association between dietary fiber intake and risk of MetS^26^. In addition, there exist the association with other nutrient include of dietary fat^27^, protein^28^, mediated role of magnesium in the metabolism of glucose^29^ and dietary insulin index^30^.

Focus on dietary patterns, not single interventions, to prevent or manage metabolic syndrome. Methods like hypothetical or exploratory approaches can identify these patterns, or a combination of both through reduced rank regression (RRR)^31–33^. Dietary patterns maintained by RRR have been demonstrated to be strongly associated with the prevalence of MetS in the general population^32^.

RRR helps identify dietary patterns impacting disease outcomes using both hypothesis-driven and exploratory data-driven methods, offering greater understanding of the links between nutrition and disease pathways^34^. We used RRR to create a dietary pattern to study the connection between dietary insulin indices and nutrients-related dietary pattern with metabolic syndrome in Iranian adults.

## Methods and materials

### Study participants

The individuals who participated in the present cross-sectional study were selected from the Hoveyzeh cohort study (HCS). The HCS is a part of the PERSIAN cohort study that has been carried out in 18 different regions of Iran, including 180,000 Iranian adults^35^. Detailed information of HCS have been reported previously^36^. The HCS is a population-based cohort study conducted in Hoveyzeh County, focusing on common chronic diseases, disorders, and risk factors of non-communicable diseases (NCDs) in the Arab ethnicity from May 2016 to August 2018. At first, a total of 10,009 adults met the inclusion criteria and were living in the Hoveyzeh area, and they all agreed to take part in the cohort study. The requirements for participation included being of Iranian heritage, between the ages of 35 and 70, and residing in Hoveyzeh for at least 9 months out of the year. These individuals were invited to the cohort center located in the Hoveyzeh region to provide data. The trained interviewers gathered data on sociodemographic characteristics, physical activity, and dietary intake. Among 10,009 participants, individuals with diabetes (n = 2226), cardio vascular diseases (n = 1704), renal failure (n = 123), gallstone disease (n = 297), chronic lung disease (n = 585), any type of cancers (n = 37), pregnancy and/or lactating (n = 163), reported daily energy intakes outside the range of 800–4200 kcal/day (n = 856)^37^ and incomplete FFQ (n = 113) were excluded.

**Figure 1:**
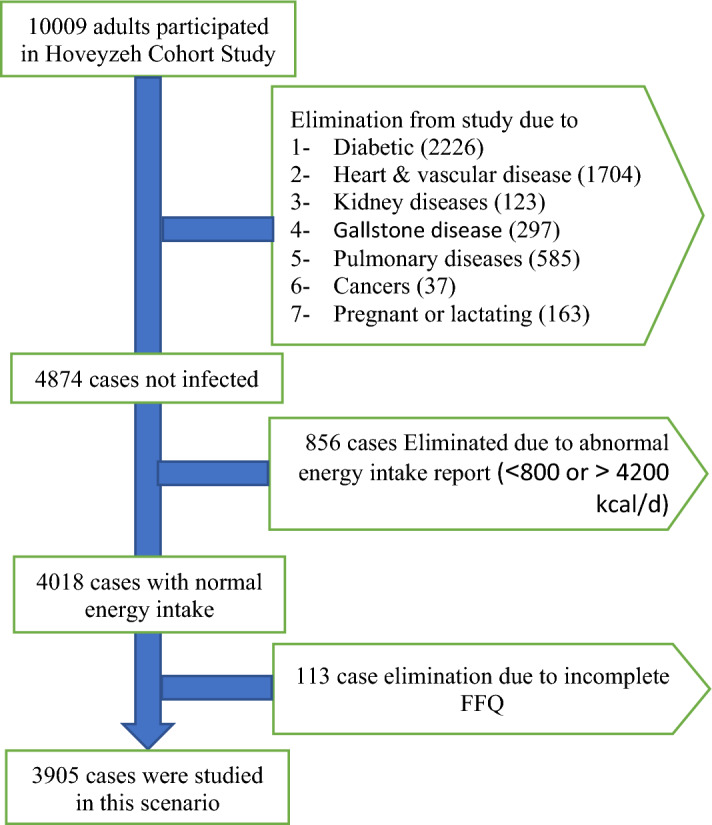
Study flow chart.

Finally, of the initial 10,009 participants, 3905 persons were included in the final analysis (Fig. 1). The Ethics Committee of Jundishapur University of Medical Sciences in Ahvaz, Iran approved the Hoveyzeh Cohort Study (IR.AJUMS.REC.1399.738). All individual participants included in the study provided informed consent.

### Biochemical and blood pressure assessment

Participants fasted for 10–12 h before collecting biological samples. Trained staff took blood, urine, hair, and nail samples immediately after enrollment. To separate serum from blood, samples were centrifuged at 1000 rpm for 15 min and stored at − 80 °C until analysis. Details of the procedure are included in the preliminary study^36^. Furthermore, we assessed Fasting Blood Sugar (FBS) utilizing the glucose oxidase technique. Enzymatic kits from Pars Azmoon in Iran were employed to determine Total Cholesterol (TC) and HDL levels. Additionally, we recorded the systolic and diastolic blood pressure of participants while seated, twice from each arm, employing standard mercury sphygmomanometers with a ten-minute interval between readings. The average of the two measurements was reported.

### Anthropometric measurements and physical activity

Trained interviewers conducted face-to-face interviews using pretested questionnaires to gather demographic variables, medical histories, medication, and smoking habits. Anthropometric data were collected in the morning after biological sampling, as this reduced measurement errors or biases. The participants' height (cm) was determined by employing a stadiometer (Seca 206), whereas their weight (kg) was measured using a standing scale (Seca 755). Additionally, their waist, hip, and wrist circumferences in centimeters were gauged using Seca locked tape meters. Following the measurements, participants were treated to breakfast before they were interviewed using a questionnaire. A Modifiable Activity Questionnaire (MAQ), which previously modified and validated among Iranians were used for physical activity measurement^38^. The participants were asked to report their frequency and duration of engagement in light, moderate, hard, and very hard activities from the past year. The activities included routine daily tasks, and the outcome was expressed in metabolic equivalent hours per week (MET-h/wk) to assess their physical activity levels.

### Dietary assessment

A 130-item Food Frequency Questionnaire (FFQ) was used to assess dietary intake over the previous year. It was divided into 29 food groups and classified as semi-quantitative. The cohort article stated that this FFQ was used for dietary assessment^36^. During the study, participants were asked to indicate their frequency of consumption for each food item, ranging from daily to yearly. The food intake was measured using household measures and then converted to grams per day^39^. The total energy and nutrient intake was calculated using Nutritionist IV.

### Insulin indices

The food insulin indices were determined by measuring the insulin response in the bloodstream after consuming each food item. This involved comparing the area under the insulin response curve for 1000 kJ (239 kcal) of each food with that of a reference food. The resulting insulin index value for each food item was derived from these measurements^40^.

We used previously reported dietary Insulin Index values to calculate total dietary II and IL^18,40,41^. We matched the food items in our FFQ with these items. For the remaining foods, we used the II of similar items based on their nutritional content correlation. To calculate the total dietary IL, we multiplied the II value of each food by its energy intake and summed the values for all items^41^. The insulin load calculated as follow: Insulin load of a given food = Insulin index of that food × energy content of that food (kcal/day). The dietary II was then obtained by dividing the dietary IL by the total energy intake^41^.

### Definition of MetS

Criteria for metabolic syndrome are abdominal obesity (waist circumference ≥ 102 in men and ≥ 88 in women), serum triglycerides (≥ 150 mg/dL), or use of hypertriglyceridemia drugs, and elevated serum levels. Reduced high density lipoprotein (HDL) cholesterol (< 40 mg/dl in men and < 50 in women) or medication for low HDL cholesterol, blood pressure ≥ 130/85 mmHg, or use of antihypertensive medications, fasting plasma glucose (FPG) ≥ 100 mg/dL or use of hyperglycemic agents^42^.

### Statistical analysis

The mean ± SD and percentages were reported for total participants and MetS based on quartiles of DII and DIL for continuous and categorical variables, respectively. One-way analysis of variance was used to evaluate differences in quantitative variables and the χ^2^ test was used to assess the distribution of categorical variables (Tables 1, 2).

**Table 1:** General characteristics of total participants across quartiles of DII and DIL scores and participants with MetS, Hoveyzeh cohort study, Khuzestan, Iran^1^.

Variables	Quartiles of DII				P-value^2^	Quartiles of DIL				P-value^2^
	1N = 950	2N = 951	3N = 951	4N = 950		1N = 950	2N = 951	3N = 951	4N = 950	
	Total participants					Total participants				
Age (year)	47.92 ± 9.27	47.28 ± 8.98	46.98 ± 8.67	46.98 ± 8.60	0.06	49.93 ± 9.85	47.20 ± 8.85	46.32 ± 8.11	45.72 ± 8.05	< 0.001
Weight (kg)	75.02 ± 15.71	75.78 ± 14.49	76.37 ± 14.76	75.57 ± 15.32	0.1	71.72 ± 15.23	75.85 ± 14.73	76.87 ± 14.82	78.70 ± 14.63	< 0.0001
Body mass index (kg/m^2^)	27.95 ± 5.26	28.11 ± 5.02	28.36 ± 5.15	28.20 ± 5.36	0.3	27.78 ± 5.52	28.48 ± 5.23	28.10 ± 5.00	28.25 ± 5.02	0.02
Hip circumference (cm)	102.89 ± 9.67	103.39 ± 9.44	103.91 ± 9.59	103.86 ± 10.24	0.07	102.72 ± 10.04	104.18 ± 9.89	103.47 ± 9.61	103.68 ± 9.37	0.01
Waist/hip ratio	0.94 ± 0.06	0.94 ± 0.06	0.94 ± 0.06	0.93 ± 0.06	0.1	0.94 ± 0.06	0.94 ± 0.06	0.94 ± 0.06	0.94 ± 0.06	0.98
FBG (mg/dl)	93.29 ± 10.25	93.91 ± 10.44	93.40 ± 9.83	93.87 ± 10.15	0.4	94.68 ± 10.73	93.71 ± 10.19	93.05 ± 9.62	93.02 ± 10.04	0.001
Diastolic blood pressure (mmHG)	70.40 ± 10.92	70.79 ± 10.97	70.35 ± 11.08	70.80 ± 10.83	0.6	70.48 ± 11.45	70.34 ± 10.65	70.20 ± 10.98	70.66 ± 10.72	0.80
Systolic blood pressure (mmHG)	111.57 ± 16.27	110.92 ± 17.36	111.16 ± 17.48	110.19 ± 16.96	0.3	111.64 ± 18.64	111.09 ± 16.09	110.29 ± 16.41	110.83 ± 16.49	0.3
Cholesterol (mg/dl)	187.43 ± 37.79	187.77 ± 36.50	187.90 ± 37.23	187.07 ± 37.35	0.9	189.74 ± 36.64	188.70 ± 39.68	185.85 ± 37.11	185.88 ± 35.14	0.04
HDL-C (mg/dl)	50.71 ± 11.98	50.29 ± 11.71	50.40 ± 12.73	51.00 ± 11.89	0.5	51.69 ± 11.43	51.05 ± 11.97	50.05 ± 12.96	49.61 ± 11.84	0.001
TG (mg/dl)	148.40 ± 91.48	155.40 ± 101.02	151.97 ± 89.95	146.41 ± 82.63	0.1	143.90 ± 78.44	148.78 ± 87.69	152.73 ± 95.40	156.77 ± 102.62	0.01
Drug anti-lipid yes (%)	5.8	6.1	6.1	6.3	0.9	5.8	6.0	6.2	6.3	0.9
Drug anti-HTN yes (%)	9.3	8.9	12	8.3	0.03	9.8	7.7	12.2	8.8	0.007
Drug anti-DM yes (%)	6.3	8.3	7.5	8.1	0.3	6.3	7.9	8.7	7.3	0.2
Physical activity (Met-h/w)	37.26 ± 5.65	37.30 ± 5.32	37.52 ± 5.33	37.73 ± 5.39	0.1	36.42 ± 4.95	37.27 ± 4.99	37.80 ± 5.67	38.33 ± 5.86	0.9
Residence type (urban) (%)	60.0	59.5	60.0	55.9	0.1	35.8	38.1	41.6	49.1	< 0.001
Education (university) (%)	8.3	8.4	8.7	7.1	0.5	5.6	9.5	8.1	9.4	< 0.001
Marital status (married) (%)	87.5	85.9	86.4	86.4	0.01	77.6	85.3	89.8	93.6	0.0001
Obesity (%)	33.7	33.4	35.3	34.8	0.3	33.8	36.4	34.4	32.7	0.008
Wealth status (rich) (%)	18.7	19.1	20.5	18.2	0.003	16.8	18.7	21.9	19.2	0.001
Smoking status (yes) (%)	22.3	16.3	15.4	17.1	< 0.001	16.00	13.9	18.80	22.30	0.0001
Alcohol use (yes) (%)	2.5	1.3	1.8	2.1	0.2	1.8	1.6	1.6	2.7	0.2

(n)	Participants with MetS (Quartiles of DII)					Participants with MetS (Quartiles of DIL)				
	249	273	267	258		302	281	243	221	
Age (year)	49.31 ± 9.04	49.18 ± 9.41	48.43 ± 8.52	48.90 ± 8.16	0.67	52.57 ± 9.41	48.59 ± 8.61	46.88 ± 7.74	46.76 ± 7.66	< **0.001**
Physical activity (Met-h/w)	36.03 ± 4.67	36.88 ± 5.34	36.76 ± 4.41	36.94 ± 4.38	0.11	35.68 ± 4.29	36.90 ± 4.96	36.30 ± 4.30	38.09 ± 5.09	< **0.001**
Weight (kg)	82.16 ± 15.20	80.90 ± 14.34	82.47 ± 14.42	81.73 ± 14.83	0.63	77.11 ± 14.75	81.70 ± 13.73	83.60 ± 14.39	86.37 ± 14.30	< **0.001**
Body mass index (kg/m^2^)	30.88 ± 4.85	30.38 ± 4.80	30.95 ± 4.90	30.68 ± 4.52	0.51	30.12 ± 5.14	31.09 ± 4.61	30.78 ± 4.55	31.00 ± 4.62	0.06
Hip circumference (cm)	107.42 ± 8.85	106.71 ± 9.06	108.14 ± 9.23	107.57 ± 9.07	0.33	105.89 ± 9.68	108.17 ± 8.86	108.03 ± 8.67	108.05 ± 8.64	**0.005**
WHR (cm)	0.97 ± 0.05	0.97 ± 0.06	0.96 ± 0.05	0.96 ± 0.06	0.22	0.97 ± 0.05	0.97 ± 0.06	0.97 ± 0.06	0.96 ± 0.05	0.79
FBG (mg/dl)	99.61 ± 11.58	100.33 ± 10.80	98.69 ± 10.74	100.48 ± 11.30	0.22	101.03 ± 10.89	99.10 ± 11.13	99.55 ± 10.87	99.19 ± 11.56	0.13
Diastolic blood pressure (mmHG)	72.85 ± 11.21	75.23 ± 12.21	73.70 ± 12.18	73.68 ± 12.09	0.14	74.05 ± 11.50	73.54 ± 11.64	72.99 ± 11.67	75.12 ± 13.19	0.26
Systolic blood pressure (mmHG)	117.16 ± 17.85	119.08 ± 19.91	118.64 ± 20.56	116.81 ± 19.81	0.47	119.19 ± 20.19	117.56 ± 18.98	116.93 ± 18.35	117.86 ± 20.82	0.57
Cholesterol (mg/dl)	196.64 ± 39.86	194.07 ± 35.57	193.54 ± 36.89	195.11 ± 37.62	0.79	196.67 ± 35.01	197.36 ± 40.35	194.38 ± 39.47	189.48 ± 34.00	0.08
HDL-C (mg/dl)	45.29 ± 10.27	45.21 ± 10.10	44.83 ± 10.69	44.95 ± 9.10	0.94	46.31 ± 9.64	45.74 ± 10.58	44.61 ± 10.11	43.01 ± 9.52	**0.001**
TG (mg/dl)	218.60 ± 115.39	213.07 ± 131.03	213.46 ± 109.55	205.43 ± 92.59	0.62	200.48 ± 91.15	250.75 ± 97.96	220.29 ± 116.28	229.41 ± 147.65	**0.01**
Drug anti-lipid yes (%)	6.0	7.7	4.5	8.1	0.7	7.6	6.0	7.0	5.4	0.6
Drug anti-HTN yes (%)	9.6	11.4	11.2	10.1	0.01	11.9	9.3	13.2	7.7	0.1
Drug anti-DM yes (%)	4.8	5.9	6.7	7.8	0.4	5.6	7.5	6.6	5.4	0.7
Residence type (urban) (%)	15.6	16.1	16.8	15.1	0.58	19.7	17.6	13.9	12.4	0.08
Education (university) (%)	1.7	1.7	1.5	1.5	0.99	1.1	2.2	1.3	1.8	< **0.001**
Marital status (married) (%)	20.5	21	22.3	21.4	**0.01**	21.7	23.4	20.3	19.9	< **0.001**
Obesity (%)	13.5	13.5	14.5	13	0.66	14.7	15.5	13.1	11.2	**0.002**
Wealth status (rich) (%)	4.9	5.2	6.3	4.3	0.71	5.4	5.6	5.2	4.4	0.07
Smoking status (yes) (%)	4.1	4.1	3.4	3.3	0.56	4.9	2.1	3.5	4.5	< **0.001**
Alcohol use (yes) (%)	0.5	0.3	0.4	0.6	0.71	0.3	0.4	0.4	0.7	0.27

*DII* Dietary insulin index, *DIL* Dietary insulin load, *MetS* Metabolic syndrome, *Met* Metabolic equivalent, *FBG* Fasting blood glucose, *HDL-C* High density lipoprotein-cholesterol, *TG* Triglyceride, *WHR* Waist hip ratio.

Bold p-values are statistically significant.

^1^Continuous values are shown as mean ± standard deviation and categorical values are shown as percent.

^2^One way ANOVA was used for continuous variables and χ2 test was used for categorical variables.

**Table 2:** Dietary intakes of total participants across quartiles of DII and DIL scores and participants with MetS, Hoveyzeh cohort study, Khuzestan, Iran^1^.

Variables	Quartiles of DII				P-value^2^	Quartiles of DIL				P-value^2^
	1	2	3	4		1	2	3	4	
Energy (kcal)	2921.29 ± 722.67	2916.99 ± 675.02	2922.00 ± 676.12	2883.81 ± 697.70	0.5	2043.33 ± 366.45	2696.24 ± 280.26	3198.19 ± 315.86	3706.26 ± 308.85	0.0001
Protein (g/day)	87.72 ± 22.89	91.27 ± 22.05	92.46 ± 22.55	90.09 ± 23.37	< 0.001	63.95 ± 12.83	83.59 ± 10.90	98.79 ± 12.59	115.21 ± 14.27	0.0001
fat (g/day)	71.34 ± 27.93	62.17 ± 21.89	60.25 ± 20.63	60.01 ± 19.95	< 0.001	46.27 ± 17.10	60.63 ± 19.66	69.85 ± 22.67	77.01 ± 21.16	0.0001
Carbohydrate (g/day)	491.50 ± 131.96	506.81 ± 122.30	510.95 ± 124.32	505.40 ± 129.62	0.006	349.58 ± 68.50	462.78 ± 54.40	553.22 ± 59.35	649.09 ± 67.98	0.001
Total fiber (g/day)	31.70 ± 9.99	32.07 ± 9.03	31.79 ± 8.95	30.69 ± 8.43	0.006	22.82 ± 6.04	29.52 ± 6.09	34.40 ± 6.73	39.51 ± 7.93	< 0.001
Calcium (mg/day)	926.63 ± 287.45	964.72 ± 272.23	991.00 ± 293.21	995.81 ± 346.17	< 0.001	676.79 ± 176.89	886.91 ± 182.25	1055.98 ± 203.12	1259.70 ± 275.40	< 0.001
Magnesium (mg/day)	385.95 ± 119.55	392.50 ± 106.54	392.00 ± 104.28	389.94 ± 103.04	0.5	274.96 ± 65.17	363.00 ± 66.33	426.81 ± 72.14	495.61 ± 82.62	< 0.001
Potassium (mg/day)	3807.59 ± 1352.17	3823.08 ± 1171.32	3822.90 ± 1132.57	3888.06 ± 1089.20	0.4	2770.85 ± 814.69	3605.62 ± 855.95	4164.22 ± 934.32	4801.62 ± 1083.87	< 0.001
Sodium (g/day)	5682.08 ± 1729.23	5834.69 ± 1758.88	5887.18 ± 1696.45	5752.19 ± 1869.84	0.05	4447.77 ± 1503.25	5431.04 ± 1350.25	6227.82 ± 1489.29	7049.58 ± 1572.13	< 0.001

With MetS (n)	249	273	267	258		302	281	243	221	
Energy (kcal)	2827.87 ± 707.79	2810.37 ± 659.81	2836.14 ± 658.52	2806.60 ± 716.47	0.95	2037.98 ± 355.43	2669.24 ± 252.78	3160.24 ± 303.75	3707.05 ± 291.91	< 0.001
Protein (g/day)	85.50 ± 22.32	88.35 ± 22.28	90.15 ± 22.18	88.88 ± 24.33	0.12	64.01 ± 12.81	83.39 ± 10.77	98.58 ± 12.52	116.16 ± 13.72	< 0.001
Total fat (g/day)	66.39 ± 24.97	58.95 ± 20.54	56.59 ± 19.58	57.80 ± 19.47	< 0.001	45.34 ± 16.00	57.28 ± 17.26	67.05 ± 20.58	74.94 ± 20.46	< 0.001
Carbohydrate (g/day)	481.91 ± 132.85	490.26 ± 119.70	500.11 ± 121.28	492.42 ± 133.38	0.44	350.54 ± 65.29	464.16 ± 53.7029	550.25 ± 59.94	653.43 ± 67.78	< 0.001
Total fiber (g/day)	31.69 ± 10.02	31.43 ± 9.25	31.64 ± 9.03	30.45 ± 8.60	0.38	23.08 ± 6.25	30.13 ± 5.92	34.47 ± 6.64	40.55 ± 8.19	< 0.001
Calcium (mg/day)	912.46 ± 280.34	939.39 ± 260.79	972.81 ± 276.41	995.02 ± 345.43	0.008	681.46 ± 167.46	904.67 ± 167.73	1056.12 ± 203.74	1282.62 ± 253.30	< 0.001
Magnesium (mg/day)	377.46 ± 109.18	381.65 ± 108.76	385.26 ± 105.52	386.00 ± 103.32	0.79	275.69 ± 61.49	365.62 ± 58.36	423.70 ± 64.54	505.31 ± 83.14	< 0.001
Potassium (mg/day)	3783.67 ± 1277.62	3734.04 ± 1176.54	3790.46 ± 1144.25	3894.07 ± 1088.16	0.45	2800.40 ± 792.58	3658.40 ± 786.01	4174.94 ± 856.60	4932.17 ± 1113.97	< 0.001
Sodium (g/day)	5622.97 ± 1806.08	5717.72 ± 1706.53	5653.26 ± 1554.78	5682.04 ± 1909.21	0.93	4462.90 ± 1569.22	5420.70 ± 1351.89	6235.56 ± 1466.16	7014.44 ± 1468.36	< 0.001

Generalized linear model was used to obtain the odds ratio (OR) and 95% confidence interval (CI) for MetS across quartiles of DIL and DII scores. The participants were categorized by gender and their DIL and DII quartiles were evaluated. The initial model accounted for age and energy consumption, while the second model incorporated body mass index (BMI) to identify links between DIL, DII, and the susceptibility to MetS that were not influenced by obesity. The third model included further adjustments for marital status, wealth score, smoking habits, and physical activity levels (met-h/w) (Table 3). Participants in the first quartiles of DIL and DII were considered as the reference group. The categories of DII and DIL were considered as an ordinal variable to calculate the trend of odds ratios across quartiles of DIL and DII.

**Table 3:** Odds ratio (95% confidence interval) for metabolic syndrome by gender across quartiles of DII and DIL scores, Howeyzeh cohort study, Khuzestan, Iran.

Models	Quartiles of DII				P-value^a^	Quartiles of DIL				P-value
	1	2	3	4		1	2	3	4	
Female n	581	600	609	641		763	667	546	455	
Crude model	1	0.87 (0.68; 1.10)	0.91 (0.72; 1.16)	1.03 (0.81; 1.32)	0.75	1	0.91 (0.72; 1.15)	1.14 (0.90; 1.45)	1.46 (1.14; 1.87)	0.002
Model 1^a^	1	0. 84 (0.66; 1.08)	0.84 (0.66; 1.08)	0.98 (0.76; 1.25)	0.87	1	0. 78 (0.58; 1.05)	0.86 (0.59; 1.27)	0.99 (0.59; 1.67)	0.99
Model 2^b^	1	0.88 (0.68; 1.13)	0.96 (0.75; 1.23)	1.07 (0.83; 1.37)	0.58	1	0.96 (0.75; 1.23)	1.29 (1.08; 1.65)	1.70 (1.31; 2.20)	< 0.001
Model 3^c^	1	0.87 (0.67; 1.12)	0.89 (0.69; 1.15)	0.98 (0.76; 1.27)	0.92	1	0.92 (0.72; 1.18)	1.21 (0.94; 1.55)	1.51 (1.16; 1.96)	0.002
Male n	369	351	342	309		187	284	405	495	
Crude model	1	0.98 (0.66; 1.46)	0.81 (0.55; 1.19)	0.84 (0.57; 1.23)	0.37	1	0.77 (0.52; 1.14)	0.86 (0.58; 1.28)	0.72 (0.49; 1.06)	0.09
Model 1^a^	1	0.97 (0.65; 1.44)	0.81 (0.55; 1.18)	0.83 (0.56; 1.22)	0.35	1	0.77 (0.47; 1.25)	0.87 (0.47; 1.60)	0.73 (0.34; 1.54)	0.41
Model 2^b^	1	0.91 (0.59; 1.40)	0.73 (0.48; 1.11)	0.76 (0.50; 1.16)	0.21	1	0.84 (0.54; 1.28)	0.84 (0.54; 1.29)	0. 83 (0.54; 1.26)	0.39
Model 3^c^	1	0.88 (0.57; 1.36)	0.69 (0.45; 1.06)	0.75 (0.49; 1.15)	0.18	1	0.83 (0.54; 1.28)	0.84 (0.54; 1.30)	0.83 (0.54; 1.28)	0.42

*DII* Dietary insulin index, *DIL* Dietary insulin load.

Bold p-values are statistically significant.

^a^Adjusted for age and energy intake.

^b^Further Adjusted for body mass index.

^c^Further Adjusted for marital status, wealth status, smoking status, and physical activity (met-h/w).

**Figure 2:**
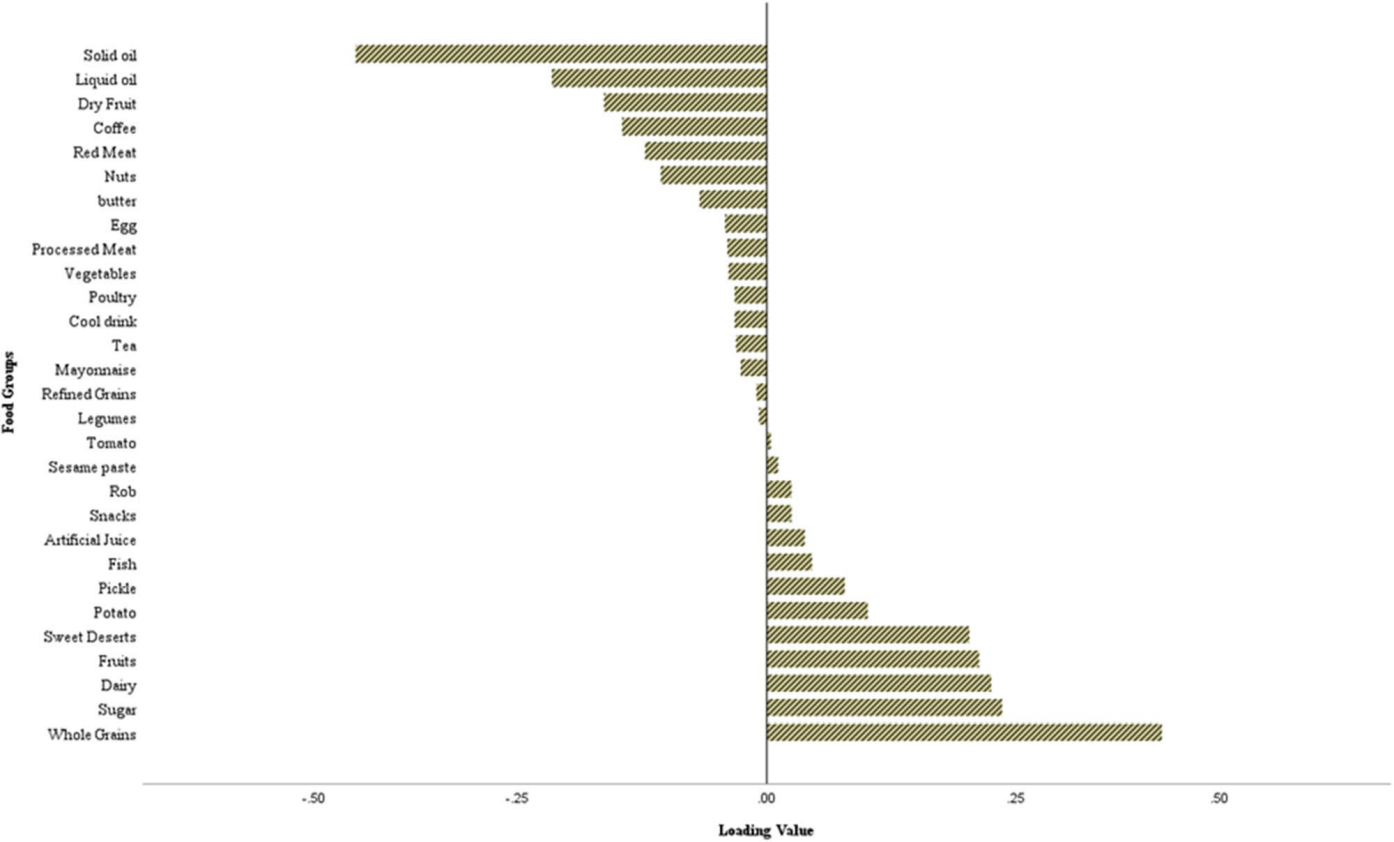
First dietary pattern loading values of all 29 food groups. Total explained variance dietary pattern is 86.2%. Explained variance for food groups is 8.6%. *Rob* traditional paste of fruits.

RRR analysis identifies dietary patterns as linear combinations of food variables that explain the variation in response variables. We used four nutrients intake, include of; fiber (g/day)^26^, magnesium (mg/day)^29^, protein, fat (g/day)^27^ and total DII^30^ as response variables, and 29 food groups as predictive variables (Supplementary Table S1). We considered the first dietary pattern for subsequent analysis, which explained the maximum total explained variance in all response variables. Food groups with factor loadings ≥ 0.11^43^ were considered to describe the dietary pattern, but all foods contributed to calculating the total score of dietary patterns. Factor loadings indicate the correlation of each food group with the dietary pattern. The dietary pattern score was obtained by summing the product of standardized intakes of the 29 food groups and their corresponding weight. The first dietary pattern was characterized by higher intake of whole grains, sugar, dairy, fruits, sweet deserts and lower intake of solid oil, liquid oil, dry fruit, coffee, red meat (Fig. 2).

The number of dietary patterns extracted by RRR was determined by the number of response variables. However, the only first dietary pattern due to achieve higher explained variance was considered for further analysis (Supplementary Table S2). Correlations between extracted dietary pattern scores and nutrients serving as response variables were assessed using Spearman correlation coefficient (Supplementary Table S3). In this study, we analyzed four essential nutrients: protein (measured in grams per day), fat (g/day), fiber (g/day), and magnesium (mg/day), in addition to the dietary insulin index. To determine the odds ratio (OR) and 95% confidence interval (CI) for MetS based on gender, we utilized a generalized linear model with three adjusted models, examining quartiles of the first dietary pattern score. As a reference point, we used the lowest quartile (Table 4). Moreover, we provided an overview of the general characteristics of all participants by gender, examining quartiles of DII and DIL scores (Supplementary Table S4). We used SAS statistical software (SAS Studio 3.71; SAS Institute Inc.) to identify the dietary patterns and SPSS version 22 (SPSS Inc, Chicago, IL) was used for all other statistical analyses. Two-sided P values < 0.05 were considered statistically significant.

**Table 4:** Odds ratio (95% confidence interval) for metabolic syndrome by gender across quartiles of dietary pattern score in whole population, Howeyzeh cohort study, Khuzestan, Iran.

Models	Quartiles of dietary pattern				P for trend
	1	2	3	4	
Female n = 2475					
Crude model	1	0.98 (0.78; 1.24)	1.04 (0.82; 1.32)	1.190 (0.93; 1.51)	0.4
Model 1^a^	1	1.01 (0.79; 1.32)	1.03 (0.81; 1.32)	1.24 (0.96; 1.57)	0.2
Model 2^b^	1	1.03 (0.80; 1.32)	1.13 (0.91; 1.39)	1.41 (1.13; 1.75)	0.01
Model 3^C^	1	1.04 (081; 1.33)	1.08 (0.84; 1.4)	1.24 (0.96; 1.60)	0.3
Male n = 1412					
Crude model	1	1.21 (0.84; 1.75)	1.19 (0.83; 1.70)	1.98 (1.33; 2.93)	0.008
Model 1^a^	1	1.21 (0.84; 1.74)	1.19 (0.83; 1.70)	1.96 (1.32; 2.92)	0.009
Model 2^b^	1	1.22 (0.81; 1.83)	1.11 (0.75; 1.65)	2.09 (1.35; 3.21)	0.006
Model 3^C^	1	1.21 (0.80; 1.81)	1.10 (0.74; 1.64)	2.08 (1.35; 3.21)	0.007

^a^Adjusted for age and energy intake.

^b^Adjusted for model 1 + body mass index.

^c^Adjusted for model 2 + marital status, wealth score, smoking status, and physical activity (met-h/w).

### Ethical approval and consent to participate

This project was approved by the research council (research project number: NRC_9906) and the ethics committee (research ethics number: IR.AJUMS.REC. 1399.738) of Ahvaz Jundishapur university of medical sciences. Participants were fully informed about the study objectives and methods, and were assured of the confidentiality of their information. An informed consent was obtained from all subjects. All methods were carried out in accordance with relevant guidelines and regulations.

## Results

The average participants' age and BMI were 47.28 ± 8.88 years and 28.15 ± 5.20 kg/m^2^, respectively. MetS was prevalent 19.03% among men and 32.3% of women. The DII average for women was significantly higher than that of men (women: 40.7 ± 4.16, men: 40.23 ± 3.84, P = 0.001). However, the DIL average for men was significantly higher than that of women (men 128,320.28 ± 27,870.13 and women 112,157.98 ± 30,715.45 P = 0.0001). General characteristics of the study population according to the quartiles of dietary DIL and DII are indicated in the Table 1.

Compared with the first quartile of DIL, Participants with MetS in the upper quartile of DIL were younger, had higher body weight and body mass index, were physically active, had higher triglyceride levels, and lower level of HDL cholesterol. Similarly, the whole population under study in the upper quartile of DIL were more likely to have higher body mass index and triglycerides. And in the same quartile, they were younger, married, had higher education level, higher income status, and more likely to smoke.

Dietary intakes of the study population based on the quartiles of DIL and DII are expressed in the Table 2. The dietary intake of whole population showed the same as Individuals with MetS in the top quartile of DIL, had higher intakes of macronutrients and micronutrients comprise of; Energy, Total Protein, fat, Carbohydrate, Total fiber, Calcium, Magnesium, Potassium and Sodium. Participant with MetS in the highest quartile of DII were more likely to have higher intake of calcium and lower intake of total fat. In addition, the whole population in the top quartile of DII showed a rise in intake of total protein, Carbohydrate and Calcium.

Table 3 shows the multivariate-adjusted ORs and 95% CIs for MetS across quartiles of DIL and DII in men and women. No significant association was seen between DIL, DII, and MetS among men. Additionally, there was no significant association between DII and MetS in women. Moreover, a significant positive association was observed between DIL and MetS after adjusting for confounding factors. These factors including age, energy intake present in Model1, BMI in addition with Model1 considered as Model2, marital status, wealth score, smoking status and physical activity in addition with Model 2 indicated as Model 3. Female participants in higher quartile of DIL showed higher weight, BMI or central obesity were mostly married. They appeared to consume more energy and other macronutrients with lower age. Although, they were most active and had lower serum levels of FBS and total cholesterol. Similarly, younger male participants in top quartile of DIL revealed higher weight and consumed more energy and other macronutrients, however were most active and married (Supplementary Table S4).

The odds ratios for MetS, when comparing the highest with the lowest quartile of DIL in crude and two significant adjusted models were (OR = 1.46; 1.14; 1.87), (OR = 1.70; 1.31; 2.20), and (OR: 1.51; 95% CI 1.16; 1.96) respectively.

Regarding RRR analysis, five dietary patterns were identified, however dietary pattern 1 (DP1) explained the greatest proportion of variance for selected nutrients with 55.3%, and total explained variance of 86.2% (Supplementary Table S2). Therefore, we considered only DP1 for subsequent analysis. DP1 was characterized by high consumption of whole grain, sugar, dairy products, fruits and sweet deserts with highest positive loading values, and low intakes of solid oil, liquid oil, dry fruits, coffee and red meat with highest negative loading values (Fig. 2).

Odds ratio (95% CI) for MetS across quartiles of dietary pattern are provided in Table 4. After adjustments for potential confounder include of age, energy intake, body mass index, marital status, wealth score, smoking status, and physical activity, female in the top quartile of the dietary pattern had 41% increased odds of MetS compared with those in the bottom quartile (OR: 1.41; 95% CI 1.13; 1.75). However, male in contrast to female elevated OR value of MetS in the highest quartile of dietary pattern.

## Discussion

Results of the present study revealed that adherence to a diet with a high insulin load (DIL) was significantly associated with an increased odds of MetS in women by 51%. No significant association was seen between dietary insulin indices and odds of MetS in men. Additionally, men and women in top quartile of DIL were younger and married, had greater consumption of energy and other macronutrients with obese BMI level. The whole population under study were younger in higher quartile of DIL with higher weight and BMI level. However, they had lower FBS, total cholesterol serum level and lower HDL level as well.

In one study, they found that higher adherence to dietary diversity was associated with higher energy intake and macronutrients. The increasing risk of having MetS in the higher dietary diversity for old female could be attributed to a higher likelihood of abdominal obesity, which could be attributed to different food consumption in higher categories of dietary diversity^44^. Eating a modern diet with pork, poultry, vegetables, seafood, pastries, and snacks can lead to higher energy intake, BMI, waist circumference, TG, and FPG for both men and women^45^.

Although the precise causes of Metabolic Syndrome (MetS) are unclear, insulin resistance and hyperinsulinemia have been shown to be involved in the etiology of Metabolic Syndrome (MetS) and its components^46^. It is well known that diet can play a pivotal role in insulin secretion. Recent observational studies have shown that diets that increase the insulin response may lead to several metabolic disorders^13,30,47^. Various methods exist to evaluate a diet's insulinemic potential, but the DIL and DII indices have gained significant attention in recent times. These indices are based on the insulin response and can accurately measure the secretion of insulin in response to foods that are rich in carbohydrates and proteins^48^. A prospective cohort study indicated that a positive association between DII and DIL with insulin resistance in men and women^49^.

The results of the Shahedieh cohort study indicate that following a diet with a high DIL and DII can increase the risk of MetS in women. Similarly, the Tehran Lipid and Glucose Study found a positive association between higher DII, DIL and MetS risk in women. Additionally, among Iranian adolescents, higher DIL and DII were strongly linked to increased risk of metabolically unhealthy obesity. Moreover, among women hs-CRP play a potential mediatory role in the association between DIL and MetS^21,23,24^.

Despite the few longitudinal study on the association of the dietary DII and DIL with MetS risk, there are several studies reported the controversial findings on the association of dietary DII or DIL with MetS risk factors, including hyperglycemia, insulin resistance, obesity, and dyslipidemia^30,47,49,50^. Results of two cohort studies showed that a diet with high DII and DIL was positively related with increased serum levels of TG and inversely associated with serum HDL-C concentrations^13^. However, this study could not find any significant relationship between the DII and DIL and the biomarkers of glycemic control (c-peptide and hemoglobin A1c). In addition, a cross-sectional study by Hajhashemy et al.^25^ revealed that the DII and DIL were strongly associated with odds of metabolically unhealthy obese. In contrast, Ghorbaninejad et al. could not find a significant relationship between DII and DIL with the risk of metabolic syndrome and obesity^22^. Likewise, in a cohort study conducted within the framework of SEPAHAN data set, Anjom-Shoae et al. showed that higher DII and DIL were not associated with the risk of abdominal obesity^30^. The reasons for these discrepancies between studies may be due to different methods for calculating DII and DIL, different food processing and cooking methods in different cultures and considering different confounders. In the present study, the significant positive relationship between DIL and MetS was only seen in women. The exact mechanism of this sex difference is unknown. However, it may be due to the influence of sex hormone levels and androgen/estrogen balance and their effect of appetite and body composition^51–53^. One potential explanation could be the lower prevalence of MetS in men compared to women, resulting in larger confidence intervals. The specific mechanisms and pathways by which DII and DIL impact MetS are currently unknown. Whilst, female subjects had 44.6% obesity this may be increased the susceptibility of MetS. Gender-associated factors corelated with genetic and biological signaling pathways are primarily determined by hyperandrogenism, insulin resistance, and associated increased abdominal obesity and decreased postmenopausal HDL cholesterol. Gender-related factors are sensitive to social and cultural behaviors, dietary habits, and psychosocial factors. Women are more likely than men to develop MstS in response to work stress and low socioeconomic status^54^. Most married women in the present study were in the highest quartile of DII and DIL. Additionally, a greater percentage of obese women were placed in the highest quartile of DIL.

Likewise another study found out women who have ever been married are more likely to be overweight or obese due to factors such as older age, wealthy households, higher education, food security, television watching, and urban unemployment^55^. Retrospective cohort showed that individuals who get married are more likely to gain weight due to changes in their eating habits after marriage, which can lead to inconsistent weight control^56^. A community-based cohort study found that women who were married had a higher likelihood of being obese and having abdominal obesity. This could be attributed to getting married leading to more frequent meals and late-night snacking, as well as the chance to eat together, which results in an overall increase in energy intake^57^.

A diet with high DII may facilitate obesity by enhancing more insulin secretion, which may reduce fat oxidation and increase carbohydrate oxidation, therefore, such a diet could increase fat storage, abdominal obesity and enhanced risk of MetS^25,58^.

Furthermore, foods that cause high insulin levels are quickly digested and absorbed, resulting in a rapid increase in blood glucose and insulin and a subsequent decrease in glucose levels^59^. This rapid decrease in blood glucose can reduce satiety, restoring hunger sensation, thus contribute to excessive calorie intake, which could lead to an increased risk of abdominal obesity and MetS^59,60^. However, all insulinemic foods with high-DII are not quickly transformed into glucose and directly enhance insulin secretion. Iranian people receive more than 55% of energy from the carbohydrates-rich foods which is considerably higher than the amounts consumed in other populations^61^. Also, the main sources of carbohydrate intake among the Iranians are refined grains such as rice, white bread and potato^62^ which are considered as the insulinogenic foods that can increased risk of insulin resistant and MetS^63^. In the current study, individuals in the fourth quartile of DIL significantly consumed more carbohydrate than those in the first quartile. Therefore, higher consumption of the insulinogenic foods or high insulin index foods including rice, white bread, and potato may play a crucial role in decreasing the MetS risk.

In the current study, five dietary patterns were identified based on RRR, but the first pattern, which explained 55.3 of the response variation, was considered as the main pattern. This pattern was characterized by higher intakes of fruits, sugar, sweet deserts, whole Grains and dairy and less intakes of solid oils, liquid oils, dry fruits, coffee and red meat. A significant positive association was found between this dietary pattern and risk of MetS. Our research aligns with a cross-sectional study of young and middle-aged Taiwanese adults with dyslipidemia and abnormal FPG. It found that those who consumed a western-style diet, high in desserts and sugary foods, were at increased risk for general obesity, central obesity, and high body fat^64^. The relation of Gender differences in the prevalence of metabolic syndrome may be due to physiological differences such as hormones, social and psychological stressors, and lifestyle differences^65^.

Regarding dietary pattern, previous studies using RRR have used different indicators as response variables to generate dietary patterns predictive of the risk of metabolic diseases. Some studies used plasma and blood biomarkers as response variables^66,67^ and others used nutrients from food as response variables^68^. The use of dietary nutrients as response variables could result in a greater variation of responses as they are more proximal in the causal chains. However, the assumption of independence for response variables may be violated when using predictors and responses from dietary assessment tools^69^. Our results in regarding the association of dietary pattern and MetS risk are comparable with those identified in previous studies. A study in Sweden found that a diet high in sugar-sweetened beverages, reduced-fat milk, artificially sweetened beverages, and sweets is linked to a higher risk of MetS^70^. A study by Asadi et al. that conducted in Iran reported a positive association between a dietary pattern rich in sugar and sweetened beverage and risk of MetS^71^. Results from a cross-sectional study in Germany revealed that a dietary pattern with greater loading on high-sugar beverages, eggs, potatoes, beer and sweets was associated with risk of MetS^72^. The effect of food groups on body weight and obesity could define their relationship with MetS, a significant risk factor^73,74^. Surprisingly, fruits and whole grains had a high load in the identified pattern. In contrast with our results, epidemiological studies indicated that diet rich in fruits and whole grains is associated with reduced risk of MetS^75–77^. This discrepancy could be explained by differences in socio-demographic characteristics, behavioral and lifestyle factors. It should be noted that in our study we used fiber and magnesium as response variables that are found in abundance in fruits and whole grains. Epidemiological evidences reported controversial findings in the relationship between dairy with MetS and its components. Some studies showed an inverse association between dairy and risk of MetS^78,79^, while others failed to show such association^80,81^. A review on the relationship between dairy fat content and risk of metabolic disease does not support the hypothesis that dairy fat or high-fat dairy foods contribute to obesity or cardiometabolic risk^82^.

The main strength of present study was the large sample size and ethnicity origin of Arab. Other strengths were the use of food-frequency and reliable and validate physical activity questionnaires and the collection of a broad range of confounding factors. However, the present study had also some limitations. First, we used the US Department of Agriculture (USDA) databank because the Iranian food composition table was not complete in some food items and micronutrients, which possibly can cause measurement error. Second, the DII of some food items in FFQ were not available in the reference list and we used the DII of similar foods. Third, our study was not prospective design and we used data of the first phase of cohort (cross-sectional data). Therefore, the results of current study should be interpreted with caution and generalizability of findings will reduce due to Arab population-based. Hence, despite considering for a wide variety of confounders in our analysis, it is not possible to confidently rule out unmeasured or unknown confounding effects.

## Conclusion

In conclusion, findings from this population-based cohort study showed that adherence to a high insulin loading diet in women was associated with greater risk of MetS. Additionally, dietary pattern derived from RRR suggested that diet rich in fruits, sugar, sweet deserts, whole Grains and dairy products were associated with increased risk of MetS. Further observational studies with long-term follow up are required to substantiate these findings.

### Supplementary Information

Supplementary Tables.

## Data Availability

The data that support the findings of this study are available from The Hoveyzeh Cohort Study (HCS), but restrictions apply to the availability of these data, which were used under license for the current study and so are not publicly available but is available with the corresponding author upon reasonable request.

## Abbreviations

BMI: Body mass index
CI: Confidence interval
CVD: Cardiovascular disease
DP: Dietary pattern
DII: Dietary Insulin Index
DIL: Insulin Load
FFQ: Food Frequency Questionnaire
FPG: Fasting plasma glucose
FBS: Fasting Blood Sugar
HCS: Hoveyzeh Cohort Study
HDL: High-density lipoprotein
LDL: Low-density lipoproteins
MAQ: Modifiable Activity Questionnaire
MET-h/wk: Metabolic equivalent hours per week
MetS: Metabolic Syndrome
OR: Odds ratio
RRR: Reduced-rank regression
TC: Total cholesterol
USDA: US Department of Agriculture
WC: Waist circumference
WHR: Waist to hip ratio

## References

[1] Grundy SM (2016). Metabolic syndrome update. Trends Cardiovasc. Med..

[2] Belete R, Ataro Z, Abdu A, Sheleme M (2021). Global prevalence of metabolic syndrome among patients with type I diabetes mellitus: A systematic review and meta-analysis. Diabetol. Metab. Syndr..

[3] Hageman PA, Pullen CH, Hertzog M, Boeckner LS, Walker SN (2012). Associations of cardiorespiratory fitness and fatness with metabolic syndrome in rural women with prehypertension. J. Obes..

[4] Fatahi A, Doosti-Irani A, Cheraghi Z (2020). Prevalence and incidence of metabolic syndrome in Iran: A systematic review and meta-analysis. Int. J. Prevent. Med..

[5] Xu H, Li X, Adams H, Kubena K, Guo S (2019). Etiology of metabolic syndrome and dietary intervention. Int. J. Mol. Sci..

[6] Xu SHQN (2016). Gender differences in dietary patterns and their association with the prevalence of metabolic syndrome among Chinese: A cross-sectional study. Nutrients..

[7] Shang X (2017). Dietary protein from different food sources, incident metabolic syndrome and changes in its components: An 11-year longitudinal study in healthy community-dwelling adults. Clin. Nutr..

[8] Carlson JJ, Eisenmann JC, Norman GJ, Ortiz KA, Young PC (2011). Dietary fiber and nutrient density are inversely associated with the metabolic syndrome in US adolescents. J. Am. Dietetic Assoc..

[9] Calton EK, James AP, Pannu PK, Soares MJ (2014). Certain dietary patterns are beneficial for the metabolic syndrome: Reviewing the evidence. Nutr. Res..

[10] Hu FB (2002). Dietary pattern analysis: A new direction in nutritional epidemiology. Curr. Opin. Lipidol..

[11] Fabiani R, Naldini G, Chiavarini M (2019). Dietary patterns and metabolic syndrome in adult subjects: A systematic review and meta-analysis. Nutrients..

[12] Sotos-Prieto M (2021). Association between the Mediterranean lifestyle, metabolic syndrome and mortality: A whole-country cohort in Spain. Cardiovasc. Diabetol..

[13] Nimptsch K (2011). Dietary insulin index and insulin load in relation to biomarkers of glycemic control, plasma lipids, and inflammation markers. Am. J. Clin. Nutr..

[14] Ang M, Müller AS, Wagenlehner F, Pilatz A, Linn T (2012). Combining protein and carbohydrate increases postprandial insulin levels but does not improve glucose response in patients with type 2 diabetes. Metabolism..

[15] Grill V, Qvigstad E (2000). Fatty acids and insulin secretion. Br. J. Nutr..

[16] Newsholme P, Bender K, Kiely A, Brennan L (2007). Amino acid metabolism, insulin secretion and diabetes. Biochem. Soc. Transact..

[17] Bao J, De Jong V, Atkinson F, Petocz P, Brand-Miller JC (2009). Food insulin index: Physiologic basis for predicting insulin demand evoked by composite meals. Am. J. Clin. Nutr..

[18] Bell KJ, Petocz P, Colagiuri S, Brand-Miller JC (2016). Algorithms to improve the prediction of postprandial insulinaemia in response to common foods. Nutrients..

[19] Teymoori F, Farhadnejad H, Moslehi N, Mirmiran P, Mokhtari E, Azizi F (2021). The association of dietary insulin and glycemic indices with the risk of type 2 diabetes. Clin. Nutr..

[20] Teymoori F, Farhadnejad H, Mirmiran P, Nazarzadeh M, Azizi F (2020). The association between dietary glycemic and insulin indices with incidence of cardiovascular disease: Tehran lipid and glucose study. BMC Public Health..

[21] Sadeghi O, Hasani H, Mozaffari-Khosravi H, Maleki V, Lotfi MH, Mirzaei M (2020). Dietary insulin index and dietary insulin load in relation to metabolic syndrome: The Shahedieh Cohort Study. J. Acad. Nutr. Dietetics..

[22] Ghorbaninejad P (2021). Higher dietary insulin load and index are not associated with the risk of metabolic syndrome and obesity in Iranian adults. Int. J. Clin. Practice..

[23] Khoshnoudi-Rad B, Hosseinpour-Niazi S, Javadi M, Mirmiran P, Azizi F (2022). Relation of dietary insulin index and dietary insulin load to metabolic syndrome depending on the lifestyle factors: Tehran lipid and glucose study. Diabetol. Metab. Syndr..

[24] Darzi M (2023). The possible mediatory role of inflammatory markers on the association of dietary insulin index and insulin load with metabolic syndrome in women with overweight and obesity: A cross-sectional study. Int. J. Clin. Pract..

[25] Hajhashemy Z, Mirzaei S, Asadi A, Akhlaghi M, Saneei P (2022). Association of dietary insulin index and dietary insulin load with metabolic health status in Iranian Overweight and Obese Adolescents. Front. Nutr..

[26] Chen JP, Chen GC, Wang XP, Qin L, Bai Y (2017). Dietary fiber and metabolic syndrome: A Meta-analysis and review of related mechanisms. Nutrients..

[27] Julibert A, Bibiloni MDM, Tur JA (2019). Dietary fat intake and metabolic syndrome in adults: A systematic review. Nutr. Metab. Cardiovasc. Dis..

[28] Campos-Nonato I, Hernandez L, Barquera S (2017). Effect of a high-protein diet versus standard-protein diet on weight loss and biomarkers of metabolic syndrome: A randomized clinical trial. Obes. Facts..

[29] Piuri G, Zocchi M, Della Porta M, Ficara V, Manoni M, Zuccotti GV (2021). Magnesium in obesity, metabolic syndrome, and type 2 diabetes. Nutrients..

[30] Anjom-Shoae J (2020). Association between dietary insulin index and load with obesity in adults. Eur. J. Nutr..

[31] Panagiotakos DBPC, Skoumas Y, Stefanadis C (2007). The association between food patterns and the metabolic syndrome using principal components analysis: The ATTICA study. J. Am. Diet. Assoc..

[32] Janett Barbaresko SS (2014). Comparison of two exploratory dietary patterns in association with the metabolic syndrome in a Northern German population. Br. J. Nutr..

[33] Kesse-Guyot E, Ahluwalia N, Lassale C, Hercberg S, Fezeu L (2013). Adherence to Mediterranean diet reduces the risk of metabolic syndrome: A 6-year prospective study. Nutr. Metab. Cardiovasc. Diseases..

[34] Hoffmann K, Schulze MB, Schienkiewitz A, Nöthlings U, Boeing H (2004). Application of a new statistical method to derive dietary patterns in nutritional epidemiology. Am. J. Epidemiol..

[35] Poustchi H, Eghtesad S, Kamangar F, Etemadi A, Keshtkar AA, Hekmatdoost A (2018). Prospective epidemiological research studies in Iran (the PERSIAN Cohort Study): Rationale, objectives, and design. Am. J. Epidemiol..

[36] Cheraghian B (2020). Cohort profile: The Hoveyzeh Cohort Study (HCS): A prospective population-based study on non-communicable diseases in an Arab community of Southwest Iran. Med. J. Islamic Republic Iran..

[37] Esmaillzadeh AAL (2008). Major dietary patterns in relation to general obesity and central adiposity among Iranian women. J. Nutr..

[38] Momenan AA, Delshad M, Sarbazi N, Rezaei Ghaleh N, Ghanbarian A, Azizi F (2012). Reliability and validity of the Modifiable Activity Questionnaire (MAQ) in an Iranian urban adult population. Arch. Iranian Med..

[39] Azar, M., Sarkisian, E. Food composition table of Iran, Vol. 65. (National Nutrition and Food Research Institute, Shaheed Beheshti University, 1980).

[40] Holt S, Miller J, Petocz P (1997). An insulin index of foods: The insulin demand generated by 1000-kJ portions of common foods. Am. J. Clin. Nutr..

[41] Bao J, Atkinson F, Petocz P, Willett WC, Brand-Miller JC (2011). Prediction of postprandial glycemia and insulinemia in lean, young, healthy adults: Glycemic load compared with carbohydrate content alone. Am. J. Clin. Nutr..

[42] Metabolic syndrome (insulin resistance syndrome or syndrome X) [Internet]. Jul 30, 2020. https://www.uptodate.com/contents/metabolic-syndrome-insulin-resistance-syndrome-or-syndrome-x.

[43] Naja F, Itani L, Hwalla N, Sibai AM, Kharroubi SA (2019). Identification of dietary patterns associated with elevated blood pressure among Lebanese men: A comparison of principal component analysis with reduced rank regression and partial least square methods. PLoS One..

[44] Tian X, Zhang K, Wang H (2017). Gender difference of metabolic syndrome and its association with dietary diversity at different ages. Oncotarget..

[45] Wang Y, Tian T, Zhang J, Xie W, Pan D, Xu D, Lu Y, Wang S, Xia H, Sun G (2021). The effects of dietary pattern on metabolic syndrome in Jiangsu Province of China: Based on a nutrition and diet investigation project in Jiangsu Province. Nutrients..

[46] Okosun IS, Okosun B, Lyn R, Airhihenbuwa C (2020). Surrogate indexes of insulin resistance and risk of metabolic syndrome in non-Hispanic White, non-Hispanic Black and Mexican American. Diabetes Metab. Syndrome Clin. Res. Rev..

[47] Mozaffari H, Namazi N, Larijani B, Surkan PJ, Azadbakht L (2019). Associations between dietary insulin load with cardiovascular risk factors and inflammatory parameters in elderly men: A cross-sectional study. Br. J. Nutr..

[48] Yari Z, Behrouz V, Zand H, Pourvali K (2020). New insight into diabetes management: From glycemic index to dietary insulin index. Curr. Diabetes Rev..

[49] Mirmiran P, Esfandiari S, Bahadoran Z, Tohidi M, Azizi F (2015). Dietary insulin load and insulin index are associated with the risk of insulin resistance: A prospective approach in Tehran lipid and glucose study. J. Diabetes Metab. Disord..

[50] Nimptsch K, Brand-Miller J, Franz M, Sampson L, Willett W, Giovannucci E (2010). Dietary insulin index and insulin load in relation to biomarkers of glycemic control, and plasma lipids. Das Gesundheitswesen..

[51] Hadaegh F, Hasheminia M, Lotfaliany M, Mohebi R, Azizi F, Tohidi M (2013). Incidence of metabolic syndrome over 9 years follow-up; the importance of sex differences in the role of insulin resistance and other risk factors. PloS One..

[52] Clegg DJ (2007). Estradiol-dependent decrease in the orexigenic potency of ghrelin in female rats. Diabetes..

[53] Pelletier G, Li S, Luu-The V, Labrie F (2007). Oestrogenic regulation of pro-opiomelanocortin, neuropeptide Y and corticotrophin-releasing hormone mRNAs in mouse hypothalamus. J. Neuroendocrinol..

[54] Pucci G, Alcidi R, Tap L, Battista F, Mattace-Raso F, Schillaci G (2017). Sex- and gender-related prevalence, cardiovascular risk and therapeutic approach in metabolic syndrome: A review of the literature. Pharmacol. Res..

[55] Sarma H (2016). Determinants of overweight or obesity among ever-married adult women in Bangladesh. BMC Obes..

[56] Liu J, Garstka MA, Chai Z, Chen Y, Lipkova V, Cooper ME (2021). Marriage contributes to higher obesity risk in China: Findings from the China Health and Nutrition Survey. Ann. Transl. Med..

[57] Lee J, Shin A, Cho S, Choi JY, Kang D, Lee JK (2020). Marital status and the prevalence of obesity in a Korean population. Obes. Res. Clin. Pract..

[58] Kahn SE, Hull RL, Utzschneider KM (2006). Mechanisms linking obesity to insulin resistance and type 2 diabetes. Nature..

[59] Hellström PM (2013). Satiety signals and obesity. Curr. Opin. Gastroenterol..

[60] Llewellyn CH, Trzaskowski M, Van Jaarsveld CH, Plomin R, Wardle J (2014). Satiety mechanisms in genetic risk of obesity. JAMA Pediatr..

[61] Heidari Z, Feizi A, Azadbakht L, Mohammadifard N, Maghroun M, Sarrafzadegan N (2019). Usual energy and macronutrient intakes in a large sample of Iranian middle-aged and elderly populations. Nutr. Dietetics..

[62] Golzarand M, Mirmiran P, Jessri M, Toolabi K, Mojarrad M, Azizi F (2012). Dietary trends in the Middle East and North Africa: An ecological study (1961–2007). Public Health Nutr..

[63] Radhika G, Van Dam RM, Sudha V, Ganesan A, Mohan V (2009). Refined grain consumption and the metabolic syndrome in urban Asian Indians (Chennai Urban Rural Epidemiology Study 57). Metabolism..

[64] Lin LY, Hsu CY, Lee HA, Tinkov AA, Skalny AV, Wang WH, Chao JC (2019). Gender difference in the association of dietary patterns and metabolic parameters with obesity in young and middle-aged adults with dyslipidemia and abnormal fasting plasma glucose in Taiwan. Nutr. J..

[65] Yi Y, An J (2020). Sex differences in risk factors for metabolic syndrome in the Korean Population. Int. J. Environ. Res. Public Health..

[66] Nettleton JA (2007). Associations between markers of subclinical atherosclerosis and dietary patterns derived by principal components analysis and reduced rank regression in the Multi-Ethnic Study of Atherosclerosis (MESA). Am. J. Clin. Nutr..

[67] Seah JY, Ong CN, Koh W-P, Yuan J-M, van Dam RM (2019). A dietary pattern derived from reduced rank regression and fatty acid biomarkers is associated with lower risk of type 2 diabetes and coronary artery disease in Chinese adults. J. Nutr..

[68] Maddock J, Ambrosini GL, Griffin JL, West JA, Wong A, Hardy R (2019). A dietary pattern derived using B-vitamins and its relationship with vascular markers over the life course. Clin. Nutr..

[69] Weikert C, Schulze MB (2016). Evaluating dietary patterns: The role of reduced rank regression. Curr. Opin. Clin. Nutr. Metab. Care..

[70] Drake I, Sonestedt E, Ericson U, Wallström P, Orho-Melander M (2018). A Western dietary pattern is prospectively associated with cardio-metabolic traits and incidence of the metabolic syndrome. Br. J. Nutr..

[71] Asadi Z, Shafiee M, Sadabadi F, Saberi-Karimian M, Darroudi S, Tayefi M (2019). Association Between dietary patterns and the risk of metabolic syndrome among Iranian population: A cross-sectional study. Diabetes Metab. Syndrome Clin. Res. Rev..

[72] Heidemann C, Scheidt-Nave C, Richter A, Mensink GB (2011). Dietary patterns are associated with cardiometabolic risk factors in a representative study population of German adults. Br. J. Nutr..

[73] Andres-Hernando A, Kuwabara M, Orlicky DJ, Vandenbeuch A, Cicerchi C, Kinnamon SC (2020). Sugar causes obesity and metabolic syndrome in mice independently of sweet taste. Am. J. Physiol.-Endocrinol. Metab..

[74] Stanhope KL (2016). Sugar consumption, metabolic disease and obesity: The state of the controversy. Crit. Rev. Clin. Lab. Sci..

[75] Cattafesta M, Salaroli LB (2018). Diets high in vegetables, fruits, cereals, and tubers as a protective factor for metabolic syndrome in bank employees. Diabetes Metab. Syndrome Obesity Targets Therapy..

[76] Wei Z-Y, Liu J-J, Zhan X-M, Feng H-M, Zhang Y-Y (2018). Dietary patterns and the risk of metabolic syndrome in Chinese adults: A population-based cross-sectional study. Public Health Nutr..

[77] Falahi E, Anbari K, Ebrahimzadeh F, Roosta S (2017). Dietary patterns associated with metabolic syndrome: The Khorramabad Study. J. Nutr. Food Security..

[78] Dugan CE, Fernandez ML (2014). Effects of dairy on metabolic syndrome parameters: A review. Yale J. Biol. Med..

[79] Bhavadharini B, Dehghan M, Mente A, Rangarajan S, Sheridan P, Mohan V (2020). Association of dairy consumption with metabolic syndrome, hypertension and diabetes in 147,812 individuals from 21 countries. BMJ Open Diabetes Res. Care..

[80] Duffey KJ, Gordon-Larsen P, Steffen LM, Jacobs DR, Popkin BM (2010). Drinking caloric beverages increases the risk of adverse cardiometabolic outcomes in the Coronary Artery Risk Development in Young Adults (CARDIA) Study. Am. J. Clin. Nutr..

[81] Beydoun MA, Gary TL, Caballero BH, Lawrence RS, Cheskin LJ, Wang Y (2008). Ethnic differences in dairy and related nutrient consumption among US adults and their association with obesity, central obesity, and the metabolic syndrome. Am. J. Clin. Nutr..

[82] Kratz M, Baars T, Guyenet S (2013). The relationship between high-fat dairy consumption and obesity, cardiovascular, and metabolic disease. Eur. J. Nutr..

