# Supplementary material for: Dietary insulin index, dietary insulin load and dietary patterns and the risk of metabolic syndrome in Hoveyzeh Cohort Study
Source: Sci Rep. 2024 Jan 23;14:1968. doi: 10.1038/s41598-024-52263-5 (PMC10806255; doi:10.1038/s41598-024-52263-5)
Supplement: Supplementary file 1 — Supplementary Tables. [file 41598_2024_52263_MOESM1_ESM.docx]

**Supplementary table S1.** Food groupings and their components

| Food group | Dietary components | |
| --- | --- | --- |
|  |  | |
| Whole grain | Barbari*, Sangak*, Taftoon*, Barley |  |
| Sugar | Sugar, sugar cube, candy, sugar candy |  |
| Dairy | Milk, yogurt, cheese, chocolate milk, cream, ice cream |  |
| Fruits | Cantaloupe, watermelon, melon, apple, orange, apricot, cherry, fig, peach, pear, citrus fruits, date, kiwi, grapes, pomegranate, strawberry, banana, grapefruit, other fruits. |  |
| Sweet dessert | Cakes, cookies, chocolate, pastry, honey, jam, halva |  |
| Potato | Potato |  |
| Pickle | \| Pickles, salted vegetables \| \| --- \| |  |
| Fish | Fish |  |
| Artificial juice | Packed juices |  |
| Snack | Biscuits, corn chips, potato chips |  |
| **Rob | Traditional Fruits extraction past |  |
| Sesame paste | sesame paste |  |
| Tomato | Tomato, tomato paste, Tomato sauce |  |
| Legumes | Bean, chickpea, split pea, soybean, lentil, kidney bean |  |
| Refine grain | Lavash*, baguette, rice, macaroni, pasta |  |
| Mayonnaise | Mayonnaise |  |
| Tea | Tea |  |
| Cool drink | Non-alcoholic drink |  |
| Poultry | Chicken |  |
| Vegetables | Carrot, spinach, lettuce, mixed vegetables, eggplant, zucchini, pumpkin, local vegetables, pepper, mushroom, cucumber, garlic, cabbages, onion, and other vegetables. |  |
|  |  |  |
| Processed meat | Sausage- hamburger- sausages, |  |
| Egg | Egg |  |
| Butter | Butter |  |
| Nuts | Peanut, almond, walnut, pistachio, hazelnut, roasted seeds |  |
| Red meat | all kinds of red meats, heart, liver, kidney, trip and rennet, brain, tongue, head sheep meat, leg sheep meat |  |
| Coffee | Coffee |  |
| Dry fruits | dried figs, dried dates, and other dried fruit |  |
| Liquid oil | Liquid oil and olive oil |  |
| Solid oil | Solid oil, margarine, Solid vegetable oil |  |
|  |  | |

* A kind of traditional bread. **traditional paste of fruits

**Supplementary table S2**. The association between dietary patterns and response variables

|  |  | Response Variables | | | | |  |
| --- | --- | --- | --- | --- | --- | --- | --- |
| Total explained variance | Current explained variance | Total fiber | magnesium | Total fat | Total protein | Dietary insulin index | Dietary patterns |
| 55.3592 | 55.3592 | 73.2134 | 85.6819 | 44.329 | 73.4292 | 0.1425 | 1 |
| 71.3556 | 15.9964 | 75.081 | 86.5637 | 75.4491 | 76.203 | 43.4812 | 2 |
| 80.2512 | 8.8956 | 84.644 | 88.9358 | 91.0583 | 78.4929 | 58.1249 | 3 |
| 84.7417 | 4.4905 | 89.1765 | 88.9804 | 93.2572 | 91.3123 | 60.982 | 4 |
| 86.2432 | 1.5015 | 91.3932 | 93.6658 | 93.2713 | 91.8949 | 60.9906 | 5 |

Reported as a percentage.

Conducting analysis with reduced rank regression (RRR).

**Supplementary table S3**. Spearman correlation between response variables and RRR-derived dietary patterns

| Response Variables | | | | |  |
| --- | --- | --- | --- | --- | --- |
| Total fiber(g/d) | Magnesium  (mg/d) | Total fat(g/d) | Total protein(g/d) | Dietary insulin index | Dietary patterns |
| 0.51 | 0.55 | 0.4 | 0.51 | 0.02 | 1 |
| 0.15 | 0.10 | -0.62 | 0.18 | 0.73 | 2 |
| -0.46 | -0.23 | 0.59 | 0.22 | 0.57 | 3 |
| 0.44 | 0.04 | 0.31 | -0.75 | 0.35 | 4 |
| 0.54 | -0.79 | 0.04 | 0.27 | -0.03 | 5 |

**Supplementary table S4.** General characteristics of total participants by gender across quartiles of DII and DIL scores Hoveyzeh cohort study, Khuzestan, Iran^1^.

| Variables | Quartiles of DII | | | | P-value^2^ | Quartiles of DIL | | | | P-value^2^ | |
| --- | --- | --- | --- | --- | --- | --- | --- | --- | --- | --- | --- |
|  | 1  N=950 | 2  N=951 | 3  N=951 | 4  N=950 |  | 1  N=950 | 2  N=951 | 3  N=951 | 4  N=950 |  |  |
|  | **Male Participants** | | | |  | **Male Participants** | | | |  |  |
| Age (y) | 48.12 ± 9.40 | 47.09 ±8.75 | 48.50 ± 9.10 | 48.32 ±8.85 | 0.1 | 50.89 ± 10.24 | 47.39±9.29 | 48.18±8.67 | 47.10±8.50 | <0.001 |  |
| Weight (kg) | 80.64 ± 15.57 | 80.30 ± 13.95 | 80.62 ±13.98 | 79.42 ±15.50 | 0.7 | 77.47 ± 14.71 | 79.38 ± 14.99 | 80.51 ± 15.23 | 81.67 ± 14.08 | 0.006 |  |
| Body mass index (kg/m^2^) | 26.77 ±4.73 | 26.76 ±4  36 | 26.86  ±4.13 | 26.58 ±4.85 | 0.8 | 26.38 ± 4.71 | 26.44± 4.49 | 26.72 ± 4.65 | 27.09 ± 4.33 | 0.1 |  |
| Hip circumference (cm) | 100.33 ± 8.95 | 100.11 ± 7.72 | 100.29 ±7.89 | 99.81 ± 9.19 | 0.8 | 99.09 ± 8.42 | 99.60 ± 8.23 | 100.03 ± 8.89 | 100.95 ± 8.14 | 0.03 |  |
| Waist circumference (cm) | 95.25±11.88 | 94.61±11.05 | 95.16±10.44 | 94.54±12.14 | 0.7 | 93.91± 11.82 | 94.20 ± 11.50 | 94.73 ± 11.84 | 95.82 ± 10.69 | 0.1 |  |
| Waist/hip ratio | 0.94 ± 0.06 | 0.94 ± 0.06 | 0.94 ± 0.05 | 0.94 ± 0.05 | 0.7 | 0.94 ± 0.06 | 0.94 ± 0.06 | 0.94 ±0.06 | 0.94 ± 0.05 | 0.8 |  |
| FBG (mg/dl) | 93.18 ± 9.59 | 93.78 ± 10.46 | 93.27 ± 9.05 | 93.36 ± 10.23 | 0.8 | 94.89 ± 10.67 | 93.80 ± 9.88 | 92.60 ± 9.17 | 93.26 ± 9.95 | 0.06 |  |
| Diastolic blood pressure (mm HG) | 72.15 ± 11.06 | 72.68 ± 10.23 | 72  33 ±11.02 | 72.00 ±10.60 | 0.8 | 74.49 ± 11.50 | 72.22 ± 9.95 | 72.05 ± 10.79 | 71.71 ± 10.74 | 0.02 |  |
| Systolic blood pressure (mm HG) | 113.42 ±16.31 | 113.27 ±15.01 | 115.16 ±17.17 | 113.76 ±15.21 | 0.3 | 117.35 ± 18.74 | 112.96 ± 13.70 | 113.49 ± 15.99 | 113.46 ± 15.90 | 0.01 |  |
| Cholesterol (mg/dl) | 184.70 ±37.78 | 187.53 ± 37.77 | 186.10 ± 35.36 | 187.14 ± 38.00 | 0.7 | 188.63 ± 38.46 | 185.62 ± 40.07 | 186.05 ± 38.04 | 186.08 ± 34.32 | 0.8 |  |
| HDL-C (mg/dl) | 46.31 ± 10.43 | 46.20 ±11.28 | 45.32 ±10.35 | 45.53 ±9.56 | 0.5 | 46.28 ± 9.49 | 46.84 ± 10.15 | 45.80 ± 11.43 | 45.18 ± 10.09 | 0.1 |  |
| TG (mg/dl) | 163.88 ±103.77 | 182.93 ±129.126 | 174.35 ±108.96 | 169.41 ± 96.90 | 0.1 | 166.96 ± 99.94 | 163.44 ± 97.08 | 171.69 ± 107. 52 | 180.77 ± 123.62 | 0.1 |  |
| Drug anti-lipid yes(%) | 5.7 | 6.8 | 5.0 | 6.1 | 0.7 | 4.8 | 6.3 | 6.2 | 5.9 | 0.9 |  |
| Drug anti-HTN yes(%) | 7.6 | 9.1 | 12.6 | 7.8 | 0.08 | 10.7 | 5.6 | 11.6 | 8.9 | 0.05 |  |
| Drug anti-DM yes(%) | 8.1 | 9.1 | 7.9 | 9.4 | 0.8 | 7 | 11.3 | 8.4 | 7.9 | 0.3 |  |
| Physical activity (Met-h/w) | 38.30 ±7.50 | 38.34 ±6.86 | 38.28 ±7.17 | 38.53 ±7.55 | 0.9 | 36.76 ± 7.20 | 38.36 ± 6.93 | 38.61 ± 7.42 | 38.75 ± 7.29 | 0.01 |  |
| Residence type (urban) (%) | 66.1 | 65 | 64.9 | 57.3 | 0.07 | 74.9 | 67.3 | 64.2 | 56.6 | 0.0001 |  |
| Education (university) (%) | 16.8 | 17.7 | 17.00 | 14.6 | 0.7 | 15.5 | 20.1 | 16.3 | 15.2 | 0.3 |  |
| Marital status (married) (%) | 97.3 | 97.7 | 97.7 | 96.8 | 0.1 | 93.6 | 97.2 | 97.5 | 98.8 | 0.05 |  |
| Obesity (%) | 22.8 | 20.2 | 21.3 | 21.00 | 0.3 | 20.3 | 20.4 | 22.00 | 21.8 | 0.4 |  |
| Wealth status (rich) (%) | 22.5 | 20.8 | 21.6 | 17.8 | 0.3 | 20.9 | 19.00 | 23.00 | 20.00 | 0.09 |  |
| Smoking status (yes) (%) | 42.5 | 31.1 | 36.3 | 40.1 | 0.01 | 40.6 | 32 | 38.3 | 38.8 | 0.1 |  |
| Alcohol use (yes) (%) | 6.5 | 3.1 | 4.7 | 5.8 | 0.1 | 8 | 4.9 | 3.5 | 5.3 | 0.1 |  |
| Energy (kcal) | 3216.70 ±628.34 | 3187.41 ±598.36 | 3181.17 ± 642.85 | 3174.15 ±625.73 | 0.8 | 2180.47 ±339.79 | 2756.86 ±285.95 | 3262.79 ± 310.18 | 3762.41 ±287.41 | 0.0001 |  |
| Protein (g/d) | 97.93 ± 21.45 | 100.82± 20.07 | 102.50 ± 21.47 | 99.08 ± 22.02 | 0.02 | 69.30 ±12.45 | 86.20 ±11.05 | 101.62 ±13.00 | 118.38 ±13.85 | 0.0001 |  |
| Total fat (g/d) | 76.05 ± 25.54 | 66.64 ±19.58 | 62.83 ±19.48 | 64.11 ±19.49 | 0.0001 | 50.62 ±17.70 | 61.91 ± 21.14 | 69.33 ± 21.53 | 76.01 ±19.49 | 0.0001 |  |
| Carbohydrate (g/d) | 544.75 ± 121.02 | 555.17 ±113.83 | 559.48 ± 119.98 | 559.98 ±118.47 | 0.2 | 368.98 ±66.76 | 471.82 ±55.99 | 566.88 ±57.22 | 661.97 ±63.85 | 0.0001 |  |
| Total fiber (g/d) | 34.04 ±9.32 | 34.29 ±8.91 | 33.69 ±8.54 | 32.39 ±8.49 | 0.03 | 23.34 ±6.08 | 29.21 ±5.88 | 34.22 ± 6.54 | 39.61 ±7.85 | 0.0001 |  |
|  | **Female Participants** | | | |  | **Female Participants** | | | |  |  |
| **(n)** | **302** | **281** | **243** | **221** |  | **302** | **281** | **243** | **221** |  |  |
| Age (y) | 47.80 ±9.19 | 47.39 ±9.12 | 46.13 ±8.30 | 46.34 ±8.41 | 0.002 | 49.69 ± 9.74 | 47.11 ± 8.66 | 44.95 ± 7.38 | 44.22 ± 7.23 | 0.0001 |  |
| Physical activity (Met-h/w) | 36.60 ±3.93 | 36.70 ±4.05 | 37.10 ±3.88 | 37.35 ±3.89 | 0.002 | 36.33 ± 4.22 | 36.81 ± 3.79 | 37.20 ± 3.79 | 37.88 ± 3.68 | 0.0001 |  |
| Weight (kg) | 72.10 ±14.88 | 73.14 ± 14.15 | 73.98 ± 14.66 | 73.71 ± 14.89 | 0.1 | 70.31 ± 15.04 | 74.35 ± 14.37 | 74.17 ± 13.92 | 75.46 ± 14.59 | 0.0001 |  |
| Body mass index (kg/m^2^) | 28.69 ±5.45 | 28.89 ±5.21 | 29.20 ±5.48 | 28.9 ±5.43 | 0.4 | 28.12 ± 5.65 | 29.36 ± 5.28 | 29.13 ± 5.02 | 29.52 ± 5.40 | 0.0001 |  |
| Waist circumference(cm) | 98.75±11.73 | 99.22±11.69 | 99.18±11.99 | 99.17±12.34 | 0.8 | 97.50±11.70 | 100.0±12.04 | 99.63±11.73 | 99.75±12.23 | 0.0001 |  |
| Hip circumference (cm) | 104.51 ±9.76 | 105.32 ±9.86 | 105.94 ±9.86 | 105.82 ±10.15 | 0.05 | 103.61 ±10.21 | 106.12 ± 9.90 | 106.02 ± 9.33 | 106.66 ± 9.33 | 0.0001 |  |
| Obesity (%) | 40.6 | 41.2 | 43.2 | 41.5 | 0.8 | 37.1 | 43.2 | 43.6 | 44.6 | 0.0001 |  |
| WHR (cm) | 0.94 ± 0.06 | 0.94 ± 0.06 | 0.93 ± 0.06 | 0.93 ± 0.06 | 0.06 | 0.94 ± 0.06 | 0.94 ± 0.06 | 0.94 ± 0.06 | 0.93 ± 0.05 | 0.2 |  |
| FBG (mg/dl) | 93.35 ± 10.65 | 93.98 ±10.44 | 93.48 ± 10.24 | 94.11 ±10.11 | 0.5 | 94.64 ± 10.75 | 93.67 ± 10.32 | 93.38 ± 9.93 | 92.76 ± 10.14 | 0.01 |  |
| Diastolic blood pressure (mm HG) | 69.29 ± 10.69 | 69.70 ±11.25 | 69.24 ±10.97 | 69.22 ±10.83 | 0.8 | 69.50 ± 11.23 | 69.54 ± 10.84 | 68 ± 10.92 | 69.51 ± 10.59 | 0.6 |  |
| Systolic blood pressure (mm HG) | 110.40 ± 16.16 | 109.54 ± 18.46 | 108.92 ± 17.26 | 108.47 ± 17.49 | 0.2 | 110.24 ± 18.29 | 110.29 ± 17.52 | 107.92 ± 16.33 | 107.97 ± 16.66 | 0.01 |  |
| Cholesterol (mg/dl) | 189.16 ± 37.72 | 187.92 ± 35.76 | 188.91 ± 38.23 | 187.03  ±37.07 | 0.7 | 190.02 ± 36.21 | 190.01 ± 39.47 | 185.70 ± 36.44 | 185.67 ± 36.04 | 0.04 |  |
| HDL-C (mg/dl) | 53.50 ± 12.07 | 52.68 ±11.30 | 53.25 ±13.06 | 53.64 ±12.01 | 0.5 | 53.01 ± 11.47 | 52.84 ± 12.25 | 53.20 ± 13.13 | 54.42 ± 11.73 | 0.1 |  |
| TG (mg/dl) | 138.56 ±81.57 | 139.30 ±75.75 | 139.40 ±74.45 | 135.33 ±72.30 | 0.7 | 138.25 ± 71.16 | 142.54 ± 82.66 | 138.68 ± 82.64 | 130.66 ± 63.82 | 0.08 |  |
| Smoking status (yes) (%) | 9.5 | 7.7 | 3.6 | 5.9 | 0.0001 | 10.0 | 6.1 | 4.4 | 4.4 | 0.0001 |  |
| Alcohol use (yes) (%) | 0 | 0.2 | 0.2 | 0.3 | 0.6 | 0.3 | 0.1 | 0.2 | 0 | 0.7 |  |
| Drug anti-lipid yes(%) | 5.9 | 5.7 | 6.7 | 6.4 | 0.8 | 6 | 5.8 | 6.2 | 6.8 | 0.9 |  |
| Drug anti-HTN yes(%) | 10.3 | 8.8 | 11.7 | 8.6 | 0.2 | 9.6 | 8.5 | 12.6 | 8.8 | 0.08 |  |
| Drug anti-DM yes(%) | 5.2 | 7.8 | 7.2 | 7.5 | 0.2 | 6.2 | 6.4 | 9 | 6.6 | 0.2 |  |
| Residence type (urban) (%) | 56.1 | 56.3 | 57.3 | 55.2 | 0.9 | 56.1 | 65.3 | 57.3 | 55.2 | 0.9 |  |
| Marital status (married) (%) | 81.2 | 79.00 | 80.1 | 81.4 | 0.01 | 73.7 | 80.2 | 84.1 | 87.9 | 0.0001 |  |
| Education (university) (%) | 2.9 | 3.0 | 4.1 | 3.4 | 0.6 | 3.1 | 4.9 | 2 | 3.1 | 0.03 |  |
| Energy (kcal) | 2733.66 ±716.39 | 2758.79 ±667.47 | 2776.46 ± 650.77 | 2743.84 ± 687.80 | 0.7 | 2009.72 ±365.12 | 2670.42 ±273.97 | 3150.28 ±311.74 | 3645.18 ±319.89 | 0.0001 |  |
| Protein (g/d) | 81.23 ± 21.38 | 85. 68± 21.24 | 86.82 ± 21.00 | 85.76 ± 22.78 | 0.0001 | 62.64 ±12.58 | 82.48 ±10.65 | 96.68 ±11.85 | 111.77 ±13.93 | 0.0001 |  |
| Total fat (g/d) | 68.35 ± 28.97 | 59.55 ± 22.75 | 58.80 ±21.12 | 58.03 ±19.88 | 0.0001 | 45.21 ±16.79 | 60.09 ±18.99 | 70.23 ±23.49 | 78.10 ±22.82 | 0.0001 |  |
| Carbohydrate (g/d) | 457.69 ± 127.47 | 478.52 ± 118.24 | 483.70 ± 118.35 | 479.09 ± 126.60 | 0.001 | 344.82 ±68.13 | 458.94 ±53.28 | 543.09 ±58.91 | 635.08 ±69.62 | 0.0001 |  |
| Total fiber (g/d) | 30.21 ±10.12 | 30.78 ±8.85 | 30.72 ±9.01 | 29.86 ± 8.28 | 0.2 | 22.70 ±6.03 | 29.65 ±6.18 | 34.53 ±6.88 | 39.40 ±8.02 | 0.0001 |  |
